# Impact of physical activity patterns and sedentary behavior on sarcopenia prevalence among adults aged 18 to 59: A cross-sectional study from NHANES

**DOI:** 10.1097/MD.0000000000044312

**Published:** 2025-09-05

**Authors:** Zhengmao Li, Jing Yang, Jie Yang, Shitong Liu, Xiaozheng Guo, Zhuang Cui

**Affiliations:** a Department of Epidemiology and Biostatistics, School of Public Health, Tianjin Medical University, Tianjin, China; b The Eco-City Hospital of Tianjin Fifth Central Hospital, Tianjin, China; c Tianjin Medical University General Hospital, Tianjin, China.

**Keywords:** NHANES, physical activity, risk factor, sarcopenia, sedentary behavior

## Abstract

Sarcopenia, a growing public health concern lacking targeted therapies, highlights the need to investigate modifiable factors like physical activity (PA) and sedentary behavior, which influence muscle health. However, most research focuses on older adults, with limited data on young and middle-aged populations. This study leverages the National Health and Nutrition Examination Survey (NHANES) data to investigate this topic in the US population aged 18 to 59 to address this critical gap. This cross-sectional study utilized data from 7869 participants in the NHANES (2011–2018) to assess associations between PA patterns, sedentary behavior, and sarcopenia risk. PA and sedentary behavior were measured via self-report, “weekend warriors” was defined as individuals who meet weekly physical activity guidelines (≥150 minutes) but exercise infrequently (<2 sessions/week), and sarcopenia was defined using the Foundation for the National Institutes of Health (FNIH) guidelines. Weighted multivariate logistic regression was used, with results presented as odds ratios (ORs) with 95% confidence intervals (CIs). Nonlinear associations were explored using restricted cubic splines. In the final analysis, 689 participants (8.76%) were classified as having sarcopenia. After adjusting the covariates, sedentary time (h/day) increased the risk of sarcopenia (OR = 1.05, 95% CI: 1.01–1.10), with a linear dose–response relationship. However, for every 1-hour increment in PA, there was a 6% reduction in the risk of sarcopenia (a linear relationship was also observed), and this negative association was more pronounced for vigorous PA (OR = 0.39, 95% CI: 0.29–0.53). Meanwhile, compared to inactive individuals, both “weekend warriors” (OR = 0.41, 95% CI: 0.23–0.72) and those with regular PA patterns (OR = 0.71, 95% CI: 0.54–0.92) were less susceptible to sarcopenia. These associations showed a potentially more significant trend in older (45–59 years) and male participants. This study identifies that PA can decrease the potential risk of sarcopenia in adults aged 18 to 59, whereas prolonged sedentary behavior increases it. Promoting population-level PA participation could serve as a preventive strategy for sarcopenia, however, additional research is necessary to confirm these findings.

## 1. Introduction

Sarcopenia is mainly manifested as the gradual loss of muscle mass, strength, and function, and is associated with adverse consequences such as the decline of physical function, the reduction of quality of life, and the increase of medical expenses.^[1–3]^ Globally, the reported prevalence of sarcopenia ranges from 8% to 36% due to variations in research criteria,^[4]^ highlighting it as a pressing public health issue that urgently requires attention.^[5,6]^ Up to now, there are no specific therapeutic drugs for sarcopenia.^[7]^ Therefore, it is very necessary to identify risk factors and take intervention measures to limit the progression of sarcopenia. Previous studies have found that the main factors that can cause sarcopenia include hormonal changes, chronic inflammation, unhealthy lifestyles, etc.^[8–10]^ Many researchers consider that consistent physical activity (PA) and minimizing sedentary behavior, as key aspects of a healthy lifestyle, might be effective in preventing sarcopenia.^[11–16]^

PA is defined as any bodily movement produced by skeletal muscles that requires an energy expenditure of more than 1.5 metabolic equivalents (METs).^[17]^ According to the guidelines of the World Health Organization (WHO), the amount of PA for adults should be at least 150 minutes of moderate-intensity aerobic PA per week; otherwise, it is considered a lack of PA.^[18]^ PA helps to improve sarcopenia, which is manifested as reducing apoptosis, reducing oxidative stress, anti-inflammation, improving insulin-glucose dynamics, increasing the quality and quantity of muscle proteins and mitochondria, skeletal muscle hypertrophy, positive neuromuscular adaptations and enhancing muscle blood supply, etc.^[19]^ Meta-analyses have confirmed that PA can reduce the incidence of sarcopenia, and patients with sarcopenia can improve their conditions through PA.^[4,12,20]^ Besides, previous studies have indicated that PA intensity is a crucial factor in sarcopenia risk reduction, with high-intensity PA being more effective at lowering this risk compared to low-intensity PA.^[21,22]^

Sedentary behavior is defined as any behavior of sitting, reclining, or lying down while awake, with an energy expenditure of ≤ 1.5 METs.^[23]^ The results of systematic reviews have shown that the longer the sedentary time is, the higher the all-cause mortality rate among the elderly will be, and the weaker their cognitive function will be, and there are relationships between sedentary behavior and metabolic syndrome, waist circumference, and overweight/obesity.^[9,24]^ In particular, it seems reasonable that sedentary behavior is related to sarcopenia, because sedentary behavior has been proven to be associated with increased levels of deep fat tissue and visceral fat, and these fat tissues and visceral fat have a catabolic effect on muscles by promoting protein degradation.^[16]^ Studies have found that for every additional hour of sedentary time, the risk of sarcopenia increases by 33%.^[25]^ There are also studies suggesting that sedentary behavior is caused by sarcopenia and is a product of the decline in muscle mass and physical function.^[25,26]^ It indicates that reducing sedentary behavior may be an important intervention target for preventing sarcopenia.

Sarcopenia is commonly seen as an advanced form of aging, and it only impacts the elderly.^[1]^ Therefore, the research on the relationship between PA or sedentary behavior and sarcopenia mainly focuses on older adults rather than younger age groups. However, contrary to this common perception, the gap between the development of sarcopenia and normal progression has been found to start early in life.^[1]^ Meanwhile, the report found that the sarcopenia-related hospitalization expenses are greater in patients younger than 65 than in those aged 65 and above.^[27]^ Consequently, the impact on young people still needs to be further explored. This study explored this topic among the US young population with the National Health and Nutrition Examination Survey (NHANES) database. Specifically, this work may answer the following questions: Are there dose–response relationships between PA time, sedentary time, and the risk of sarcopenia? How do the effects of different intensities of PA and sedentary time on sarcopenia differ? Are there differences in the associations among PA, sedentary behavior, and the risk of sarcopenia by age and gender? The present study could offer insights for formulating more targeted PA and sedentary guidelines for adults, making them highly significant for both scientific research and the practical early prevention of sarcopenia.

## 2. Material and methods

### 2.1. Data source and participants

**Figure 1.**
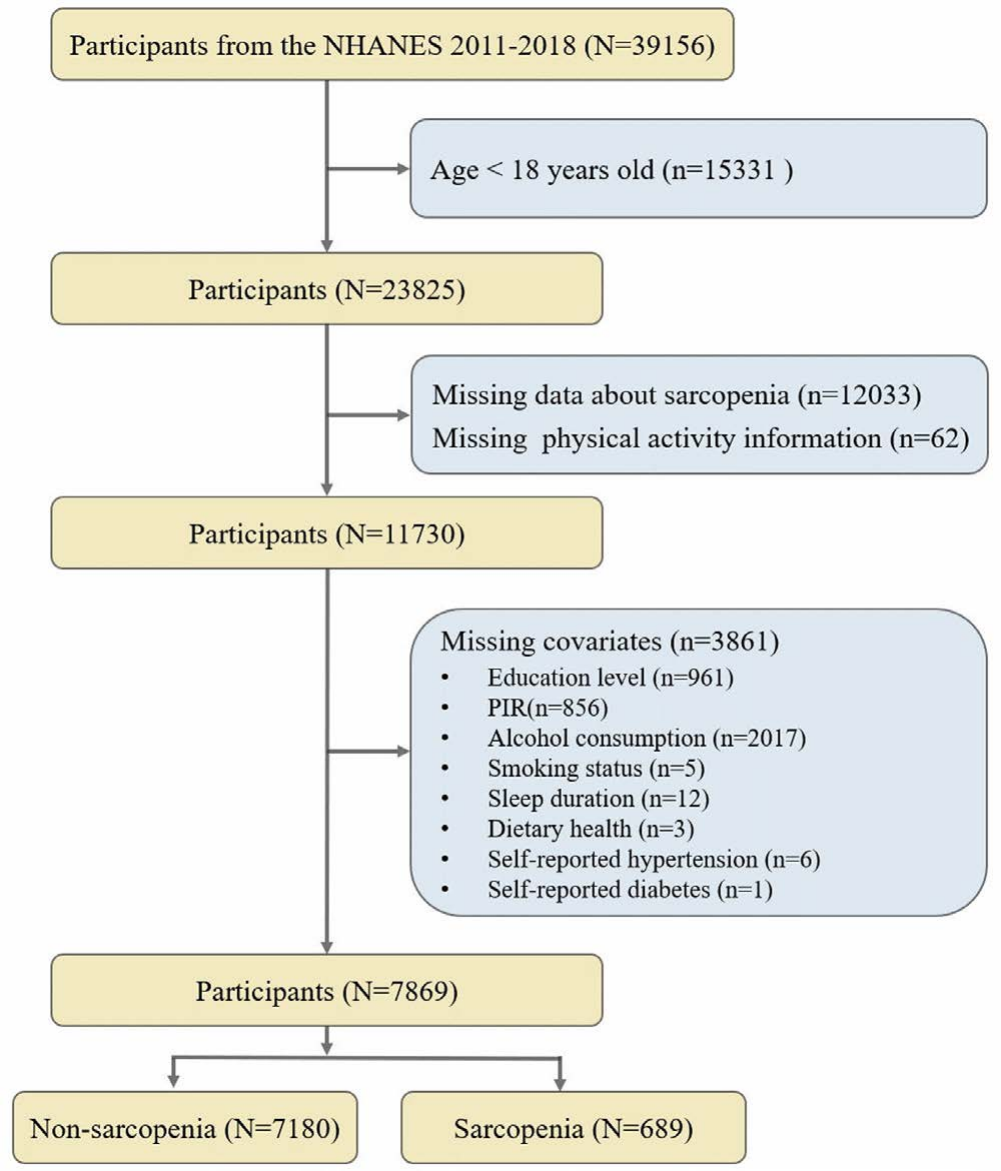
Flowchart of participant selection process: NHANES, 2011–2018. NHANES = National Health and Nutrition Examination Survey.

A total of 39,156 participants from the NHANES (2011–2018) were preliminarily included, with exclusions for aged < 18 years old (n = 15,331), limited sarcopenia data (n = 12,033), incomplete PA information (n = 62), and missing covariates (n = 3861) (Fig. 1). The final analysis includes participants with complete information (n = 7869). The National Center for Health Statistics (NCHS) Institutional Review Board has granted ethical clearance for NHANES, ensuring that all participants gave their written informed consent before engaging in the study. This research is a retrospective analysis that excludes any personally identifiable information, thereby obviating the need for further institutional review board approval for secondary analyses.

### 2.2. Sarcopenia definition

Sarcopenia, as defined by the Foundation for the National Institutes of Health (FNIH) guidelines, assesses muscle mass adequacy using the ratio of appendicular skeletal muscle mass to body mass index (ASM/BMI).^[28–30]^ Specifically, individuals are classified as sarcopenic if they have an ASM/BMI below 0.512 for females and 0.789 for males.^[28]^ In NHANES, ASM is measured using dual-energy X-ray absorptiometry (DXA) in the trunk region, as outlined by the Hologic APEX software. This measurement includes individuals aged 8 to 59 years, excluding pregnant individuals, those with a height exceeding 195.6 cm, and those weighing more than 204.1 kg. Therefore, the participants in this study were aged 18 to 59 years.

### 2.3. PA and sedentary behavior definition

PA levels were evaluated using a self-reported questionnaire based on the Global Physical Activity Questionnaire (GPAQ). The categorization of sedentary time was determined by the length of time spent in sedentary behaviors: mild sedentary behavior was considered <6 hours daily, whereas severe sedentary behavior exceeded 6 hours daily.^[31]^ The questionnaire determined PA based on participants’ engagement in vigorous or moderate recreational activities. The total PA time was calculated by summing the product of the frequency and duration for double the intensity of heavy PA, as well as the product of the frequency and duration for moderate PA.^[32,33]^ The greater frequency of heavy versus moderate PA was determined as the frequency of activity. Based on the WHO recommendations, individuals were classified as inactive if they engaged in <150 minutes of total PA per week. Those who exceeded 150 minutes but engaged in fewer than 2 sessions were labeled as “weekend warriors,” while those who surpassed 150 minutes and participated in more than 3 sessions were deemed as regularly active.^[18]^

### 2.4. Covariates definition

The following demographic factors were adjusted for as potential confounding variables: age (continuous), sex (male and female), ethnicity (Mexican American, non-Hispanic Black, non-Hispanic White, and other/multiracial), education level (less than high school graduate, high school graduate or general educational development (GED), some college and above), marital status (married/living with partner, widowed/divorced/ separated, and never married), and family poverty income ratio (PIR, continuous). Additionally, lifestyle and behavioral factors were considered as confounders in this study, including alcohol consumption, smoking status, sleep duration, and dietary habits. For the alcohol consumption questionnaire, individuals who had consumed fewer than 12 drinks in their lifetime were classified as never drinkers. Participants who had consumed alcohol but reported zero consumption over the past 12 months were deemed as former drinkers, while those who reported any consumption were considered current drinkers. Smoking status was categorized as former, current, or never based on responses to the questions: “Have you smoked at least 100 cigarettes in your life?” and “ Do you now smoke cigarettes?.” Sleep duration and dietary health information were obtained through questionnaire responses. Hypertension and diabetes were defined as conditions that had been diagnosed by medical professionals. Cardiovascular disease was identified based on self-reported occurrences of congestive heart failure, coronary heart disease, angina pectoris, heart attack, and stroke. A positive response to any of these inquiries indicated the presence of cardiovascular disease.

### 2.5. Statistical analyses

Following the NHANES Data Use Guidelines, apply sample weights (WTMEC2YR) to account for the complex survey design. Any missing data necessary for the study were excluded. Initially, we delineated and contrasted the baseline characteristics between the sarcopenia and non-sarcopenia groups. For continuous variables (such as age, PIR, and sleep duration) that did not adhere to a normal distribution, the Wilcoxon rank sum test was employed. Conversely, categorical variables were analyzed using the chi-square test.

Subsequently, to explore the associations between PA, sedentary behavior, and sarcopenia, we conducted weighted logistic regression analyses to estimate odds ratios (ORs) and 95% confidence intervals (CIs) employing 3 distinct models. Model 1 served as an unadjusted, crude model, while Model 2 incorporated adjustments for age and sex. Model 3 underwent further adjustment by including all covariates, namely ethnicity, education level, marital status, PIR, alcohol consumption, smoking status, sleep duration, dietary health, self-reported hypertension, self-reported diabetes, and self-reported cardiovascular disease. To investigate the potential nonlinear relationships, we utilized a restricted cubic splines (RCS) model. During this process, we selected the number of knots with the smallest Akaike information criterion (AIC, an indicator of statistical model fit) value, following the recommended range of 3 to 5 knots. The minimum AIC value in these models corresponded to 3 knots, which were set at the 10th, 50th, and 90th percentiles of the distribution.

Furthermore, we conducted subgroup analyses categorized by age (<45 years and ≥45 years) and sex (male and female), and explored their interactions with PA patterns and sedentary behavior on sarcopenia risk. Likelihood ratio tests compared nested models with/without interaction terms, generating *P*-values to evaluate interaction significance. To further examine the combined effects of varying levels of PA and sedentary behavior on sarcopenia risk, we conducted a joint association analysis using the lowest level of PA and severe sedentary behavior as the reference group. To evaluate the robustness of our results, we chose propensity score matching (PSM) for sensitivity analysis. In PSM analysis, we used the nearest neighbor matching method, the caliper value was 0.02, and the matching ratios employed were 1:1, 1:2, and 1:3, respectively. The standardized mean difference (SMD) was used to evaluate the quality of PSM, and an SMD value <0.1 was considered acceptable. The matching variables were all covariates. Conditional logistic regression was used to assess the associations.

Statistical significance was set at *P* < .05 for all 2-tailed tests. All analyses were executed utilizing R software (Version 4.3.3; R Foundation for Statistical Computing, Vienna, Austria).

## 3. Results

### 3.1. Participants characteristics

The participant screening process is illustrated in Figure 1. Ultimately, the study comprised a total of 7869 participants. Of these, 689 (representing 8.76%) were classified in the sarcopenia group, while 7180 (91.24%) belonged to the non-sarcopenia group, aged from 18 to 59 years old. Table 1 displays the demographic profiles of participants with and without sarcopenia. Compared to the non-sarcopenia group, individuals with sarcopenia were significantly older (median age: 39 vs 45 years), had a 2.72-fold higher proportion of Mexican Americans, exhibited lower educational levels (some college or above: 66.87% vs 51.86%), reported poorer economic status (PIR: 2.95 vs 1.97), consumed less alcohol (never: 10.58 vs 20.06), adhered to less healthy dietary habits (fair/poor: 28.70 vs 41.58), and had higher proportions of self-reported hypertension (22.34 vs 31.20), diabetes (5.20 vs 14.17), and cardiovascular disease (2.84 vs 8.56). There was also a significant difference in sleep duration between the 2 groups, although the median was 7 hours for both. Notably, no statistically significant differences were observed between the 2 groups in terms of sex, marital status, or smoking status (*P*-value > .05).

**Table 1:** Characteristics of participants in NHANES, 2011–2018.

Characteristic	Overall (n = 7869)	Non-sarcopenia (n = 7180)	Sarcopenia (n = 689)	*P*-value
Age, yr; Median (Q25%, Q75%)	40.00 (29.00, 50.00)	39.00 (29.00, 49.00)	45.00 (33.00, 54.00)	<.001
Sex, n (%)				
Male	3954 (50.85)	3614 (50.49)	340 (55.25)	.080
Female	3915 (49.15)	3566 (49.51)	349 (44.75)	
Ethnicity, n (%)				
Mexican American	1099 (9.54)	874 (8.45)	225 (22.95)	<.001
Non-Hispanic Black	1651 (11.01)	1611 (11.64)	40 (3.26)	
Non-Hispanic White	2937 (63.84)	2732 (64.75)	205 (52.60)	
Other or multiracial	2182 (15.61)	1963 (15.16)	219 (21.19)	
Education level, n (%)				
less than high school graduate	1360 (12.85)	1155 (12.11)	205 (21.98)	<.001
high school graduate or GED	1736 (21.40)	1558 (21.02)	178 (26.16)	
some college or above	4773 (65.75)	4467 (66.87)	306 (51.86)	
Marital status, n (%)				
married/living with partner	4690 (62.60)	4252 (62.78)	438 (60.38)	.052
widowed/divorced/separated	1083 (12.89)	970 (12.54)	113 (17.18)	
never married	2096 (24.51)	1958 (24.68)	138 (22.43)	
PIR; Median (Q25%, Q75%)	2.86 (1.35, 5.00)	2.95 (1.39, 5.00)	1.97 (1.03, 3.82)	<.001
Alcohol consumption, n (%)				
Former	1077 (12.63)	940 (12.08)	137 (19.44)	<.001
Current	5641 (76.08)	5255 (77.34)	386 (60.50)	
Never	1151 (11.29)	985 (10.58)	166 (20.06)	
Smoking status, n (%)				
Former	1319 (19.01)	1184 (18.71)	135 (22.68)	.072
Current	1811 (22.34)	1694 (22.67)	117 (18.28)	
Never	4739 (58.65)	4302 (58.62)	437 (59.04)	
Sleep duration, h; Median (Q25%, Q75%)	7.00 (6.00, 8.00)	7.00 (6.00, 8.00)	7.00 (6.00, 8.00)	.013
Dietary health, n (%)				
Excellent/very good	1987 (26.84)	1879 (27.61)	108 (17.28)	<.001
Good	3311 (43.49)	3048 (43.68)	263 (41.15)	
Fair/poor	2571 (29.67)	2253 (28.70)	318 (41.58)	
Self-reported hypertension, n (%)				
No	6010 (76.99)	5534 (77.66)	476 (68.80)	<.001
Yes	1859 (23.01)	1646 (22.34)	213 (31.20)	
Self-reported diabetes, n (%)				
No	7286 (94.13)	6701 (94.80)	585 (85.83)	<.001
Yes	583 (5.87)	479 (5.20)	104 (14.17)	
Self-reported cardiovascular disease, n (%)				
No	7572 (96.73)	6936 (97.16)	636 (91.44)	<.001
Yes	297 (3.27)	244 (2.84)	53 (8.56)	

Non-normal continuous variables are represented by weighted median (weighted Q25%, weighted Q75%), and categorical variables are represented by n (weighted %).

GED = general educational development, NHANES = National Health and Nutrition Examination Survey, PIR = family poverty income ratio.

### 3.2. Associations between PA, sedentary behavior, and sarcopenia risk

**Figure 2.**
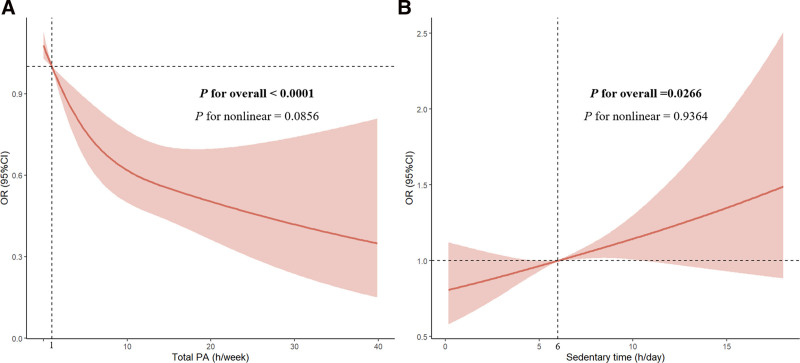
Associations between the PA (A), sedentary time (B), and sarcopenia, NHANES, 2011–2018. RCS logistic regression was used after adjusting for age, sex, ethnicity, education level, marital status, PIR, alcohol consumption, smoking status, sleep duration, dietary health, self-reported hypertension, self-reported diabetes, and self-reported cardiovascular disease. During the RCS process, we selected the knot configuration with the minimum Akaike information criterion (AIC) value within the recommended range of 3 to 5 knots. The final model used 3 knots placed at the 10th, 50th, and 90th percentiles of the exposure distribution. The solid red lines represented the central estimates, and the light red-shaded regions represented the 95% CIs. CI = confidence interval, NHANES = National Health and Nutrition Examination Survey, OR = odd ratio, PA = physical activity, PIR = family poverty income ratio, RCS = restricted cubic spline.

The potential associations between PA, sedentary behavior, and the prevalence of sarcopenia are shown in Table 2. In the unadjusted crude model and the model adjusted for age and sex, no significant association was observed between sedentary time (h/d) and sarcopenia. However, after fully adjusting for all the covariates, extended sedentary time was associated with an increased risk of sarcopenia, with an OR of 1.05 (95% CI: 1.01–1.10). When sedentary time was categorized, the association was not statistically significant. With the evolution from the crude model to a fully adjusted model, the strength of the association between PA and sarcopenia gradually weakened due to the influence of covariates. Compared to individuals who never engage in or have only light PA, those who participate in vigorous PA had a reduced risk of sarcopenia, with an OR of 0.39 (95% CI: 0.29–0.53). Additionally, for every additional hour of total PA per week, the risk of sarcopenia decreased by 6%. Moreover, compared to inactive individuals, both “weekend warriors” (OR = 0.41, 95% CI: 0.23–0.72) and those with regular PA patterns (OR = 0.71, 95% CI: 0.54–0.92) were less likely to develop sarcopenia. Linear relationships were also observed between total PA time, sedentary time, and the risk of sarcopenia (*P*-value for overall < 0.05, *P*-value for nonlinear > .05, Fig. 2). Moreover, compared to individuals with never/light PA and severe sedentary time, those with moderate PA and mild sedentary time, vigorous PA and severe sedentary time, and vigorous PA and mild sedentary time demonstrated 42%, 74%, and 59% reductions in sarcopenia risk, respectively (Table S1, Supplemental Digital Content, https://links.lww.com/MD/P857).

**Table 2:** Weighted logistic regression analyses of associations between physical activity, sedentary behavior, and sarcopenia, NHANES, 2011–2018.

Variables	Non-sarcopenia (n = 7180)	Sarcopenia (n = 689)	Model 1*		Model 2†		Model 3‡	
			OR (95% CI)	*P*-value	OR (95% CI)	*P*-value	OR (95% CI)	*P*-value
Sedentary time, h/d	6.00 (4.00, 9.00)	6.00 (4.00, 8.00)	1.00 (0.97–1.04)	.830	1.00 (0.97–1.04)	.856	**1.05 (1.01–1.10**)	**.015**
Sedentary time, n (%)								
Mild	4345 (57.87)	446 (58.92)	Reference		Reference		Reference	
Severe	2835 (42.13)	243 (41.08)	0.96 (0.74–1.24)	.739	0.95 (0.73–1.23)	.672	1.25 (0.92–1.69)	.145
Total PA time, h/wk	1.67 (0.00, 6.00)	0.00 (0.00, 2.93)	**0.91 (0.89–0.94**)	**<.001**	**0.92 (0.89–0.95**)	**<.001**	**0.94 (0.91–0.97**)	**<.001**
PA, n (%)								
Never/light	3100 (39.25)	408 (58.64)	Reference		Reference		Reference	
Moderate	1767 (26.93)	180 (27.46)	**0.68 (0.52–0.90**)	**.007**	**0.68 (0.52–0.89**)	**.006**	0.81 (0.61–1.07)	.132
Vigorous	2313 (33.82)	101 (13.90)	**0.28 (0.21–0.36**)	**<.001**	**0.30 (0.23–0.39**)	**<.001**	**0.39 (0.29–0.53**)	**<.001**
PA patterns, n (%)								
Inactive	4212 (55.68)	506 (72.43)	Reference		Reference		Reference	
Weekend warrior	554 (7.93)	24 (3.47)	**0.34 (0.19–0.60**)	**<.001**	**0.36 (0.20–0.64**)	**<.001**	**0.41 (0.23–0.72**)	**.003**
Regularly active	2414 (36.39)	159 (24.10)	**0.51 (0.40–0.64**)	**<.001**	**0.55 (0.43–0.70**)	**<.001**	**0.71 (0.54–0.92**)	**.012**

Non-normal continuous variables are represented by weighted median (weighted Q25%, weighted Q75%), and categorical variables are represented by n (weighted %). Bold values indicate statistical significance.

95 % CI = 95 % confidence interval, NHANES = National Health and Nutrition Examination Survey, OR = odds ratio, PA = physical activity, PIR = family poverty income ratio.

* Model 1: unadjusted model.

† Model 2: adjusted for age and sex.

‡ Model 3: adjusted for age, sex, ethnicity, education level, marital status, PIR, alcohol consumption, smoking status, sleep duration, dietary health, self-reported hypertension, self-reported diabetes, and self-reported cardiovascular disease.

### 3.3. Subgroup and sensitivity analysis

**Figure 3.**
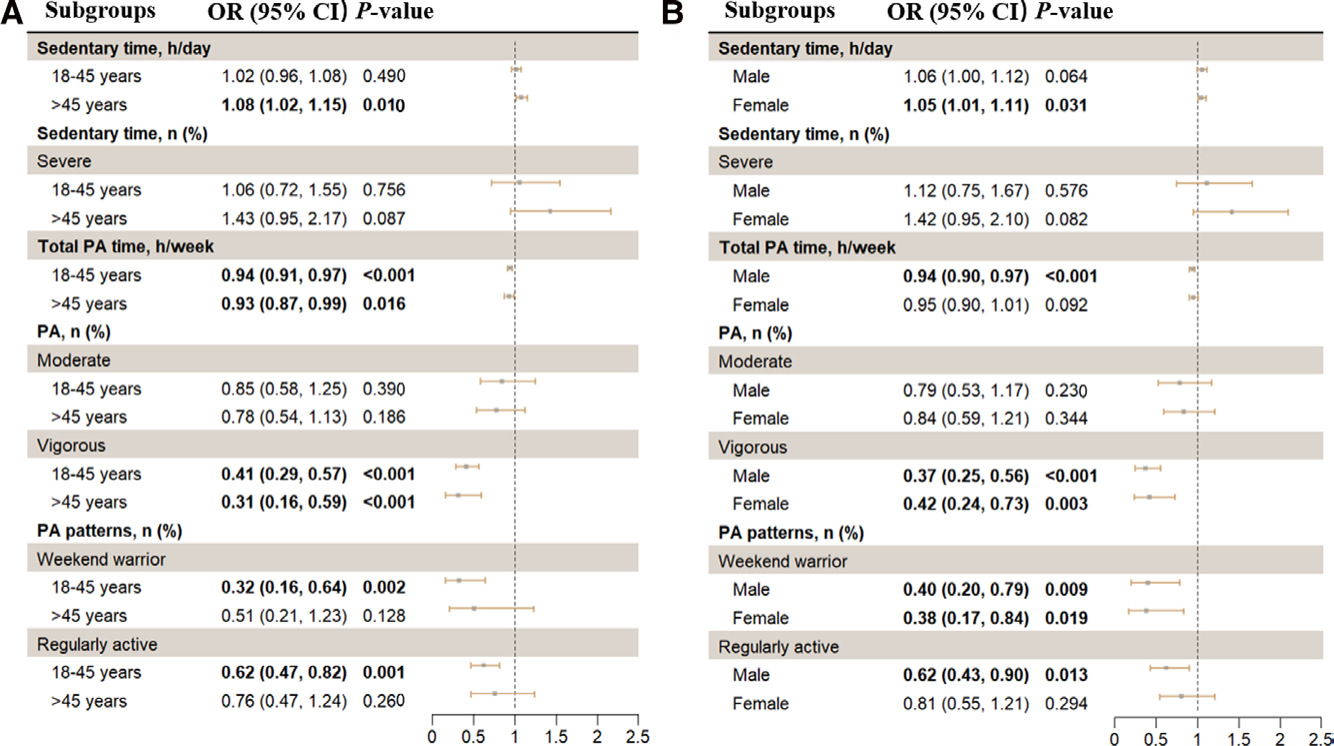
Subgroup analysis of associations between PA, sedentary behavior, and sarcopenia, NHANES, 2011–2018. Weighted logistic regression was used after adjusting for age, sex, ethnicity, education level, marital status, PIR, alcohol consumption, smoking status, sleep duration, dietary health, self-reported hypertension, self-reported diabetes, and self-reported cardiovascular disease. (A) Subgroup analysis by age. (B) Subgroup analysis by sex. CI = confidence interval, NHANES = National Health and Nutrition Examination Survey, OR = odd ratio, PA = physical activity, PIR = family poverty income ratio.

The subgroup analysis results are presented in Figure 3. An interaction analysis was performed to assess whether age and sex modify the association between PA, sedentary time, and sarcopenia risk. However, no significant interactions were observed (*P* for interaction > .05 for all comparisons), indicating that the effects of PA and sedentary time on sarcopenia risk do not differ substantially by age or sex (Fig. 3 and Table S2, Supplemental Digital Content, https://links.lww.com/MD/P857). From the results of subgroup analysis, overall, associations between PA, sedentary behavior, and risk of sarcopenia might be more pronounced in older (≥45 years) individuals and males (Fig. 3).

In addition, sensitivity analyses were performed using PSM, with detailed information provided in Table S3 (Supplemental Digital Content, https://links.lww.com/MD/P857). The results of the SMD for PSM are presented in Figure S1 (Supplemental Digital Content, https://links.lww.com/MD/P856). After PSM (1:1, 1:2, and 1:3 matching ratios), the SMDs of all covariates were <0.1, indicating adequate balance across all matched variables. In comparison to individuals who were never or only lightly active, engaging in vigorous PA was still associated with a reduced risk of sarcopenia across 3 matching iterations.

## 4. Discussion

This study used the data from the NHANES (2011–2018) to explore the relationship between PA, sedentary behavior, and the risk of sarcopenia among younger adults in the United States. The results showed that an increase in sedentary time was associated with an increased risk of sarcopenia, while an increase in PA time could reduce the risk of sarcopenia. A systematic review also demonstrated that physical inactivity and sedentary behavior are highly correlated with a decline in skeletal muscle strength and a weakening of muscle power, both of which constitute essential traits of sarcopenia.^[34]^ In contrast to inactive and sedentary subjects, physically active and non-sedentary individuals are likely to possess thicker muscle fibers, more efficient motor-unit recruitment, a greater stroke volume, an enhanced muscle oxygen consumption rate, and a more favorable hormonal balance that all together contribute to the prevention of the age-related decline in physical capabilities that is commonly observed.^[17]^

Our study showed that people who participated in vigorous PA (VPA) have a lower risk of developing sarcopenia compared with those who have never participated in it or only engaged in light physical activity (LPA). The findings suggest that insufficient PA might elevate the risk of sarcopenia, which is in line with earlier literature reviews.^[12,35–37]^ Besides, the results of this study support the evidence that PA is negatively correlated with the prevalence of sarcopenia, strengthening the unique role of VPA in reducing sarcopenia, and indicating that increasing the level of PA can effectively protect muscle mass and prevent sarcopenia.^[12]^ As per a cross-sectional study,^[38]^ individuals with greater levels of PA tend to possess an odds ratio for sarcopenia that is nearly halved, emphasizing the significance of PA as a crucial determinant in diminishing the likelihood of developing sarcopenia. However, the relationship between LPA and sarcopenia remains unclear.^[39,40]^ Likely, low-intensity activities like slow walking and light household chores do not generate sufficient physiological stimuli to maintain muscle strength in adults. Nevertheless, it should be pointed out that LPA has been proven to influence other health outcomes, such as cardiovascular diseases, cognitive health, and mortality rates, and it helps convey the public health message that “something is better than nothing.”^[41–43]^ This study also found that for every additional hour of total PA per week, the risk of sarcopenia decreased by 6%, indicating that even a slight increase in the level of PA is sufficient to significantly reduce the prevalence of sarcopenia. Previous studies have also demonstrated that a greater accumulation of self-reported PA has a protective effect on sarcopenia.^[44,45]^ However, recent studies show that intensive recreational PA reduces sarcopenia risk, with no benefit from occupational PA, and while reaching a certain PA intensity (vigorous or moderate) lowers sarcopenia risk, extremely intensive PA may not offer additional benefits.^[46]^ The possible reasons are muscle impairment and growth restriction from overexercising, as well as physical and mental stress induced by high-labor-intensity PA; the lack of autonomy and inflexible working hours associated with such activities may negatively affect muscle function, causing individuals to be unable to maintain sufficient muscle mass.^[47]^ Therefore, further research is needed to clarify the impact of different types of PA on sarcopenia and develop corresponding interventions to reduce occupational damage.

In this study, as sedentary time increases, there is a significant upward trend in the odds of developing sarcopenia. Another study has shown that compared with the group with a sedentary duration of 0 to <4 hours per day, the group with a sedentary duration of 11 hours or more per day has 2.14 times higher odds of developing sarcopenia.^[48]^ Besides, for every additional hour of sedentary time per day, the odds of developing sarcopenia will increase by 1.06 times.^[48]^ Several plausible pathways can explain the observed association. Firstly, sitting for long periods can increase the level of chronic low-grade inflammation and increase deep fat tissue and visceral fat.^[16]^ This has been proven to promote muscle atrophy and eventually stimulate protein catabolism while inhibiting muscle anabolism.^[49]^ Secondly, prolonged sedentary behavior may lead to a weakened muscle protein synthesis response by reducing the sensitivity of muscle anabolism.^[50]^ The decreased sensitivity of muscles to anabolic signals may be an important factor contributing to muscle loss and decline in physical function.^[50]^ Finally, an increase in sedentary time will replace the time for PA, thereby increasing the risk of developing sarcopenia. In addition, studies and our results all found that individuals who engage in moderate amounts of moderate-to-vigorous PA have a lower likelihood of probable sarcopenia, even if they spend a significant amount of time sedentary; conversely, those with low sedentary time do not experience a reduced risk of probable sarcopenia when their PA levels are also low.^[13]^ Longitudinal studies are needed to confirm these findings, yet they imply that efforts to prevent sarcopenia should emphasize increasing PA rather than decreasing sedentary behavior. However, studies have shown that in the group using self-reported questionnaires, sedentary behavior significantly increased the risk of sarcopenia by 66%, which is almost 16 times that of the group measured by objective physical devices (4%).^[16]^ This discrepancy may be attributed to recall bias, which leads to incorrect estimation of sedentary time compared with objective measurements, thereby strengthening the association between sedentary behavior and sarcopenia.^[51,52]^ Therefore, objective measurement tools for sedentary behavior are essential for future research on the influencing factors of sarcopenia.

In this study, we observed that associations between PA, sedentary behavior, and risk of sarcopenia might be more pronounced in older adults (≥45 years). This challenges the conventional focus on elderly populations (≥65 years) and highlights a critical window for early intervention.^[53]^ This finding is expected to facilitate a paradigm shift from geriatric-centered approaches to comprehensive lifespan prevention. A cross-sectional study investigated age-related factors linked to sarcopenia.^[54]^ Physical inactivity, metabolic risks, and low serum vitamin D levels influenced all age groups, while osteoarthropathy, fall history, and difficulties with daily activities were only related to individuals over 65.^[54]^ In the 20 to 39 and 40 to 64 age brackets, total energy intake and psychological factors emerged as associated variables.^[54]^ This result suggests that the factors affecting sarcopenia vary between young people and the elderly. The key to managing secondary sarcopenia lies in addressing its root causes, therefore, it is also of clinical significance to determine how these causative factors affect muscle function differently across various age groups. Although the prevalence of sarcopenia among young patients is lower compared to older adults, the extended duration of the condition in younger individuals could lead to more severe health complications over time.^[55]^ Therefore, further research is needed to enable early identification of sarcopenia in young people and to develop age-specific diagnostic and treatment interventions.

This study found that there might be sex differences in the significant associations among PA, sedentary behavior, and the risk of sarcopenia (although *P* for interaction > .05). A study found that the risk of sarcopenia in females is 1.9 times that in males.^[56]^ The epidemiological data on the prevalence of sarcopenia in males and females are contradictory. This may be because the pathophysiological mechanisms of sarcopenia in men and women are different.^[57]^ Menopause is a risk factor for sarcopenia in women, as the decrease in estrogen levels may lead to the deterioration of bone density, muscle mass, and muscle strength, thus contributing to the development of sarcopenia.^[58]^ In addition, the relationship between PA levels and the risk of sarcopenia may vary by sex. Insufficient PA is an important risk factor for sarcopenia in both elderly men and women, but PA levels that exceed the guidelines may significantly prevent sarcopenia in women.^[59]^ Previous studies have revealed that males and those with relatively higher education levels seem to benefit more from PA or have a lower risk of sarcopenia.^[46,60]^ However, certain demographic factors may modify the relationship between PA and sarcopenia. For example, females are more likely to engage in PA during their leisure time, while males with low incomes, who are closely associated with those with low educational attainment, mainly engage in manual labor.^[46,47]^ Therefore, future research should include individuals with diverse sociodemographic characteristics to further validate the findings.

Our observations may be of great significance in dealing with the aging of the population, these findings emphasize the importance of a healthy lifestyle (engaging in PA and limiting sedentary behavior) in preventing sarcopenia in younger adults. Previous studies have also reported that nutrition (such as protein intake, omega-3 fatty acids, and fruit and vegetable intake) is an important factor in improving muscle strength and function in adults.^[61–64]^ Therefore, in addition to advising adults to engage in PA and limit sedentary behavior, we should also recommend that adults consume 300 to 500g of vegetables and 200 to 350g of fruits daily, along with 1.0 to 1.2g of protein per kg of body weight, prioritizing high-quality sources like eggs, fish, and soy products, to improve their health conditions and prevent the occurrence of sarcopenia.^[65]^ Policymakers should implement integrated strategies combining public education, urban infrastructure upgrades, and healthcare system improvements by incorporating sarcopenia screenings into basic medical insurance. Since there are no effective treatments to reverse the progression of the disease, early prevention of sarcopenia is of utmost importance. The findings of our study could serve as a basis for developing more targeted prevention strategies against sarcopenia during early adulthood.

Some limitations should be acknowledged in our study. Firstly, this study adopted a cross-sectional design, which made it impossible to establish a causal relationship between PA, sedentary behavior, and the risk of sarcopenia. This association may be bidirectional. Future longitudinal studies are needed to test these hypotheses. Secondly, this study used self-reported questionnaires to measure the behaviors of participants and the risk of sarcopenia, which may lead to potential risks of recall bias or social desirability bias. Therefore, future studies should consider using objective measurements to verify our findings. Thirdly, in our study, the definition of sarcopenia used the FNIH standard, which was defined for the elderly population, so measurement bias might be introduced. However, our results still suggest that greater attention to sarcopenia in younger adults is warranted. Meanwhile, future research should establish youth-specific diagnostic criteria through longitudinal cohort studies to refine the sarcopenia definition. Fourthly, dietary factors, particularly protein intake, are essential for muscle repair and growth. The absence of data on protein intake may distort the relationships among PA, sedentary behavior, and sarcopenia. Future research should include comprehensive dietary evaluations to gain a better understanding of the combined impact of lifestyle and diet on sarcopenia. Finally, this study’s findings are limited to the US population, restricting global applicability due to demographic and healthcare system disparities across countries; additionally, NHANES’ exclusion of institutionalized individuals (e.g., hospitalized or nursing home residents) further limits generalizability to less healthy or functionally dependent subgroups.

## 5. Conclusions

In conclusion, in this cross-sectional study, we demonstrated a positive association between sedentary behavior and sarcopenia in US adults aged 18 to 59, and a negative association between PA and sarcopenia. It is noteworthy that both “weekend warrior” and regular PA modes of exercise are meaningful in reducing the risk of sarcopenia. Our findings offer a crucial theoretical foundation for targeted sarcopenia prevention. However, it is imperative to conduct longitudinal studies for validation and delve deeper into the biological mechanisms.

## Acknowledgments

We extend our gratitude to all participants, investigators, and academics for their invaluable contributions to the National Health and Nutrition Examination Survey.

## Author contributions

**Conceptualization:** Zhengmao Li, Jing Yang, Jie Yang, Shitong Liu, Xiaozheng Guo, Zhuang Cui.

**Formal analysis:** Zhengmao Li, Jing Yang.

**Investigation:** Zhengmao Li, Jing Yang, Jie Yang.

**Methodology:** Zhengmao Li, Jing Yang, Zhuang Cui.

**Supervision:** Zhengmao Li, Shitong Liu, Xiaozheng Guo.

**Writing – original draft:** Zhengmao Li, Jing Yang.

**Writing – review & editing:** Zhuang Cui.

## Supplementary Material



## References

[1] Cruz-Jentoft AJ Bahat G Bauer J; Writing Group for the European Working Group on Sarcopenia in Older People 2 (EWGSOP2), and the Extended Group for EWGSOP2. Sarcopenia: revised European consensus on definition and diagnosis. Age Ageing. 2019;48:16–31. 30312372 10.1093/ageing/afy169 PMC6322506

[2] Yoshimura Y Wakabayashi H Yamada M Kim H Harada A Arai H. Interventions for treating sarcopenia: a systematic review and meta-analysis of randomized controlled studies. J Am Med Dir Assoc. 2017;18:553.e1–553.e16. 10.1016/j.jamda.2017.03.019 28549707

[3] Groessl EJ Kaplan RM Rejeski WJ. Physical activity and performance impact long-term quality of life in older adults at risk for major mobility disability. Am J Prev Med. 2019;56:141–6. 30573142 10.1016/j.amepre.2018.09.006 PMC6309909

[4] Petermann-Rocha F Balntzi V Gray SR. Global prevalence of sarcopenia and severe sarcopenia: a systematic review and meta-analysis. J Cachexia Sarcopenia Muscle. 2022;13:86–99. 34816624 10.1002/jcsm.12783 PMC8818604

[5] Yuenyongchaiwat K Akekawatchai C. Prevalence and incidence of sarcopenia and low physical activity among community-dwelling older Thai people: a preliminary prospective cohort study 2-year follow-up. PeerJ. 2022;10:e13320. 35480559 10.7717/peerj.13320 PMC9037122

[6] Hoang DK Doan MC Le NM Nguyen HG Ho-Pham LT Nguyen TV. Prevalence of and risk factors for sarcopenia in community-dwelling people: the Vietnam Osteoporosis study. J Cachexia Sarcopenia Muscle. 2024;15:380–6. 38146138 10.1002/jcsm.13383 PMC10834338

[7] Kwak JY Kwon KS. Pharmacological interventions for treatment of sarcopenia: current status of drug development for sarcopenia. Ann Geriatr Med Res. 2019;23:98–104. 32743297 10.4235/agmr.19.0028 PMC7370765

[8] Liguori I Russo G Aran L. Sarcopenia: assessment of disease burden and strategies to improve outcomes. Clin Interv Aging. 2018;13:913–27. 29785098 10.2147/CIA.S149232 PMC5957062

[9] Zhang Y Liu X Ma Y Li X. Physical activity, sedentary behavior, fruit and vegetable consumption, and sarcopenia in older Chinese adults: a cross-sectional study. Nutrients. 2023;15:3417. 37571354 10.3390/nu15153417 PMC10420903

[10] Damluji AA Alfaraidhy M AlHajri N. Sarcopenia and cardiovascular diseases. Circulation. 2023;147:1534–53. 37186680 10.1161/CIRCULATIONAHA.123.064071 PMC10180053

[11] Seo JH Lee Y. Association of physical activity with sarcopenia evaluated based on muscle mass and strength in older adults: 2008-2011 and 2014-2018 Korea National Health and Nutrition Examination Surveys. BMC Geriatr. 2022;22:217. 35296249 10.1186/s12877-022-02900-3 PMC8928682

[12] Steffl M Bohannon RW Sontakova L Tufano JJ Shiells K Holmerova I. Relationship between sarcopenia and physical activity in older people: a systematic review and meta-analysis. Clin Interv Aging. 2017;12:835–45. 28553092 10.2147/CIA.S132940 PMC5441519

[13] Johansson J Morseth B Scott D Strand BH Hopstock LA Grimsgaard S. Moderate-to-vigorous physical activity modifies the relationship between sedentary time and sarcopenia: the Tromsø Study 2015-2016. J Cachexia Sarcopenia Muscle. 2021;12:955–63. 34060236 10.1002/jcsm.12718 PMC8350215

[14] García-Llorente AM Casimiro-Andújar AJ Linhares DG De Souza Vale RG Marcos-Pardo PJ. Multidomain interventions for sarcopenia and cognitive flexibility in older adults for promoting healthy aging: a systematic review and meta-analysis of randomized controlled trials. Aging Clin Exp Res. 2024;36:47. 38386173 10.1007/s40520-024-02700-2 PMC10884056

[15] Lee SY Tung HH Liu CY Chen LK. Physical activity and sarcopenia in the geriatric population: a systematic review. J Am Med Dir Assoc. 2018;19:378–83. 29580886 10.1016/j.jamda.2018.02.003

[16] Mo Y Zhou Y Chan H Evans C Maddocks M. The association between sedentary behaviour and sarcopenia in older adults: a systematic review and meta-analysis. BMC Geriatr. 2023;23:877. 38124026 10.1186/s12877-023-04489-7 PMC10734096

[17] Raffin J Rolland Y Aubertin-Leheudre M; INSPIRE group. Cross-sectional interactive associations of physical activity and sedentary behaviour with physical capacity across adulthood. J Cachexia Sarcopenia Muscle. 2024;15:1134–45. 38638004 10.1002/jcsm.13457 PMC11154759

[18] Bull FC Al-Ansari SS Biddle S. World Health Organization 2020 guidelines on physical activity and sedentary behaviour. Br J Sports Med. 2020;54:1451–62. 33239350 10.1136/bjsports-2020-102955 PMC7719906

[19] Leon AS. Attenuation of adverse effects of aging on skeletal muscle by regular exercise and nutritional support. Am J Lifestyle Med. 2017;11:4–16. 30202306 10.1177/1559827615589319 PMC6124840

[20] Beaudart C Zaaria M Pasleau F Reginster JY Bruyère O. Health outcomes of sarcopenia: a systematic review and meta-analysis. PLoS One. 2017;12:e0169548. 28095426 10.1371/journal.pone.0169548 PMC5240970

[21] Park HY Jung WS Kim SW Lim K. Relationship between sarcopenia, obesity, osteoporosis, and cardiometabolic health conditions and physical activity levels in korean older adults. Front Physiol. 2021;12:706259. 34290624 10.3389/fphys.2021.706259 PMC8287569

[22] Wu F Wills K Laslett LL Oldenburg B Jones G Winzenberg T. Moderate-to-vigorous physical activity but not sedentary time is associated with musculoskeletal health outcomes in a cohort of australian middle-aged women. J Bone Miner Res. 2017;32:708–15. 27805281 10.1002/jbmr.3028

[23] Tremblay MS Aubert S Barnes JD; SBRN Terminology Consensus Project Participants. Sedentary Behavior Research Network (SBRN) – terminology consensus project process and outcome. Int J Behav Nutr Phys Act. 2017;14:75. 28599680 10.1186/s12966-017-0525-8 PMC5466781

[24] Falck RS Davis JC Liu-Ambrose T. What is the association between sedentary behaviour and cognitive function? A systematic review. Br J Sports Med. 2017;51:800–11. 27153869 10.1136/bjsports-2015-095551

[25] Gianoudis J Bailey CA Daly RM. Associations between sedentary behaviour and body composition, muscle function and sarcopenia in community-dwelling older adults. Osteoporos Int. 2015;26:571–9. 25245026 10.1007/s00198-014-2895-y

[26] Reid N Keogh JW Swinton P Gardiner PA Henwood TR. The association of sitting time with sarcopenia status and physical performance at baseline and 18-month follow-up in the residential aged care setting. J Aging Phys Act. 2018;26:445–50. 29032697 10.1123/japa.2017-0204

[27] Sousa AS Guerra RS Fonseca I Pichel F Ferreira S Amaral TF. Financial impact of sarcopenia on hospitalization costs. Eur J Clin Nutr. 2016;70:1046–51. 27167668 10.1038/ejcn.2016.73

[28] Cawthon PM Peters KW Shardell MD. Cutpoints for low appendicular lean mass that identify older adults with clinically significant weakness. J Gerontol A Biol Sci Med Sci. 2014;69:567–75. 24737559 10.1093/gerona/glu023 PMC3991141

[29] Kim KM Jang HC Lim S. Differences among skeletal muscle mass indices derived from height-, weight-, and body mass index-adjusted models in assessing sarcopenia. Korean J Intern Med. 2016;31:643–50. 27334763 10.3904/kjim.2016.015 PMC4939509

[30] Studenski SA Peters KW Alley DE. The FNIH sarcopenia project: rationale, study description, conference recommendations, and final estimates. J Gerontol A Biol Sci Med Sci. 2014;69:547–58. 24737557 10.1093/gerona/glu010 PMC3991146

[31] Xu H Deng K Lin Z. The effects of physical activity and sedentary behavior in the associations between cardiovascular diseases and depression: a four-way decomposition. J Affect Disord. 2020;275:194–201. 32734908 10.1016/j.jad.2020.07.017

[32] Lei L Li J Wang W. The associations of “weekend warrior” and regularly active physical activity with abdominal and general adiposity in US adults. Obesity (Silver Spring). 2024;32:822–33. 38374722 10.1002/oby.23986

[33] Wang Y Nie J Ferrari G Rey-Lopez JP Rezende LFM. Association of physical activity intensity with mortality: a national cohort study of 403 681 US adults. JAMA Intern Med. 2021;181:203–11. 33226432 10.1001/jamainternmed.2020.6331 PMC7684516

[34] Ramsey KA Rojer AGM D’Andrea L. The association of objectively measured physical activity and sedentary behavior with skeletal muscle strength and muscle power in older adults: a systematic review and meta-analysis. Ageing Res Rev. 2021;67:101266. 33607291 10.1016/j.arr.2021.101266

[35] Golabi P Gerber L Paik JM Deshpande R de Avila L Younossi ZM. Contribution of sarcopenia and physical inactivity to mortality in people with non-alcoholic fatty liver disease. JHEP Rep. 2020;2:100171. 32964202 10.1016/j.jhepr.2020.100171 PMC7490851

[36] Hämäläinen O Tirkkonen A Savikangas T Alén M Sipilä S Hautala A. Low physical activity is a risk factor for sarcopenia: a cross-sectional analysis of two exercise trials on community-dwelling older adults. BMC Geriatr. 2024;24:212. 38424514 10.1186/s12877-024-04764-1 PMC10905947

[37] Shen Y Shi Q Nong K. Exercise for sarcopenia in older people: a systematic review and network meta-analysis. J Cachexia Sarcopenia Muscle. 2023;14:1199–211. 37057640 10.1002/jcsm.13225 PMC10235889

[38] Ko YC Chie WC Wu TY Ho CY Yu WR. A cross-sectional study about the relationship between physical activity and sarcopenia in Taiwanese older adults. Sci Rep. 2021;11:11488. 34075104 10.1038/s41598-021-90869-1 PMC8169879

[39] Scott D Johansson J Gandham A Ebeling PR Nordstrom P Nordstrom A. Associations of accelerometer-determined physical activity and sedentary behavior with sarcopenia and incident falls over 12 months in community-dwelling Swedish older adults. J Sport Health Sci. 2021;10:577–84. 34088651 10.1016/j.jshs.2020.01.006 PMC8500807

[40] Sánchez-Sánchez JL Mañas A García-García FJ. Sedentary behaviour, physical activity, and sarcopenia among older adults in the TSHA: isotemporal substitution model. J Cachexia Sarcopenia Muscle. 2019;10:188–98. 30920779 10.1002/jcsm.12369 PMC6438335

[41] Ekelund U Tarp J Steene-Johannessen J. Dose-response associations between accelerometry measured physical activity and sedentary time and all cause mortality: systematic review and harmonised meta-analysis. BMJ. 2019;366:l4570. 31434697 10.1136/bmj.l4570 PMC6699591

[42] Johnson LG Butson ML Polman RC. Light physical activity is positively associated with cognitive performance in older community dwelling adults. J Sci Med Sport. 2016;19:877–82. 26922133 10.1016/j.jsams.2016.02.002

[43] Ballin M Nordström P Niklasson J Nordström A. Associations of objectively measured physical activity and sedentary time with the risk of stroke, myocardial infarction or all-cause mortality in 70-year-old men and women: a prospective cohort study. Sports Med. 2021;51:339–49. 33063268 10.1007/s40279-020-01356-y PMC7846506

[44] Mijnarends DM Koster A Schols JM. Physical activity and incidence of sarcopenia: the population-based AGES-Reykjavik Study. Age Ageing. 2016;45:614–20. 27189729 10.1093/ageing/afw090 PMC5027639

[45] Tyrovolas S Koyanagi A Olaya B. Factors associated with skeletal muscle mass, sarcopenia, and sarcopenic obesity in older adults: a multi-continent study. J Cachexia Sarcopenia Muscle. 2016;7:312–21. 27239412 10.1002/jcsm.12076 PMC4864288

[46] Zhao W Dai C Wang Q. Sarcopenia risk in U.S. younger adults: the impact of physical activity intensity and occupational engagement-insights from a cross-sectional NHANES study. BMC Public Health. 2024;24:3179. 39543540 10.1186/s12889-024-20665-9 PMC11566085

[47] Jacob L Gyasi RM Oh H. Leisure-time physical activity and sarcopenia among older adults from low- and middle-income countries. J Cachexia Sarcopenia Muscle. 2023;14:1130–8. 36872652 10.1002/jcsm.13215 PMC10067478

[48] Smith L Tully M Jacob L. The association between sedentary behavior and sarcopenia among adults aged ≥65 years in low- and middle-income countries. Int J Environ Res Public Health. 2020;17:1708. 32151034 10.3390/ijerph17051708 PMC7084450

[49] Bano G Trevisan C Carraro S. Inflammation and sarcopenia: A systematic review and meta-analysis. Maturitas. 2017;96:10–5. 28041587 10.1016/j.maturitas.2016.11.006

[50] Shad BJ Wallis G van Loon LJ Thompson JL. Exercise prescription for the older population: the interactions between physical activity, sedentary time, and adequate nutrition in maintaining musculoskeletal health. Maturitas. 2016;93:78–82. 27338978 10.1016/j.maturitas.2016.05.016

[51] Chastin SFM Dontje ML Skelton DA; Seniors USP team. Systematic comparative validation of self-report measures of sedentary time against an objective measure of postural sitting (activPAL). Int J Behav Nutr Phys Act. 2018;15:21. 29482617 10.1186/s12966-018-0652-x PMC5828279

[52] Cleland C Ferguson S Ellis G Hunter RF. Validity of the International Physical Activity Questionnaire (IPAQ) for assessing moderate-to-vigorous physical activity and sedentary behaviour of older adults in the United Kingdom. BMC Med Res Methodol. 2018;18:176. 30577770 10.1186/s12874-018-0642-3 PMC6303992

[53] McGowan LJ Chater AM Harper JH. Acceptability of a remotely delivered sedentary behaviour intervention to improve sarcopenia and maintain independent living in older adults with frailty: a mixed-methods study. BMC Geriatr. 2024;24:820. 39394560 10.1186/s12877-024-05385-4 PMC11468285

[54] Bae EJ Kim YH. Factors affecting sarcopenia in Korean adults by age groups. Osong Public Health Res Perspect. 2017;8:169–78. 28781939 10.24171/j.phrp.2017.8.3.03 PMC5525561

[55] Jung HN Jung CH Hwang YC. Sarcopenia in youth. Metabolism. 2023;144:155557. 37080353 10.1016/j.metabol.2023.155557

[56] Yen HY Lee SC Lin CF Lai HR Yamaguchi Y Lee PH. Prevalence of sarcopenia and its association with diet and physical activity in older adults with type 2 diabetes: a cross-sectional study. Nurs Health Sci. 2023;25:628–35. 37783469 10.1111/nhs.13055

[57] Hwang J Park S. Gender-specific risk factors and prevalence for sarcopenia among community-dwelling young-old adults. Int J Environ Res Public Health. 2022;19:7232. 35742480 10.3390/ijerph19127232 PMC9223381

[58] Geraci A Calvani R Ferri E Marzetti E Arosio B Cesari M. Sarcopenia and menopause: the role of estradiol. Front Endocrinol (Lausanne). 2021;12:682012. 34093446 10.3389/fendo.2021.682012 PMC8170301

[59] Tsai CH Liao Y Chang SH. Cross-sectional association of physical activity levels with risks of sarcopenia among older Taiwanese adults. BMC Geriatr. 2024;24:560. 38937702 10.1186/s12877-024-05087-x PMC11210145

[60] Cheng L Sit JWH Chan HYL. Sarcopenia risk and associated factors among Chinese community-dwelling older adults living alone. Sci Rep. 2021;11:22219. 34782685 10.1038/s41598-021-01614-7 PMC8593165

[61] Robinson S Granic A Cruz-Jentoft AJ Sayer AA. The role of nutrition in the prevention of sarcopenia. Am J Clin Nutr. 2023;118:852–64. 37657521 10.1016/j.ajcnut.2023.08.015 PMC10636259

[62] Ji S Baek JY Go J. Effect of exercise and nutrition intervention for older adults with impaired physical function with preserved muscle mass (functional sarcopenia): a randomized controlled trial. Clin Interv Aging. 2025;20:161–70. 39990981 10.2147/CIA.S494781 PMC11846533

[63] Yoshimura Y Matsumoto A Inoue T Okamura M Kuzuya M. Protein supplementation alone or combined with exercise for sarcopenia and physical frailty: a systematic review and meta-analysis of randomized controlled trials. Arch Gerontol Geriatr. 2025;131:105783. 39955964 10.1016/j.archger.2025.105783

[64] Huang YH Chiu WC Hsu YP Lo YL Wang YH. Effects of omega-3 fatty acids on muscle mass, muscle strength and muscle performance among the elderly: a meta-analysis. Nutrients. 2020;12:3739. 33291698 10.3390/nu12123739 PMC7761957

[65] Chen LK Arai H Assantachai P. Roles of nutrition in muscle health of community-dwelling older adults: evidence-based expert consensus from Asian Working Group for Sarcopenia. J Cachexia Sarcopenia Muscle. 2022;13:1653–72. 35307982 10.1002/jcsm.12981 PMC9178363

