# Supplementary material for: Impact of physical activity patterns and sedentary behavior on sarcopenia prevalence among adults aged 18 to 59: A cross-sectional study from NHANES
Source: Medicine (Baltimore). 2025 Sep 5;104(36):e44312. doi: 10.1097/MD.0000000000044312 (PMC12419358; doi:10.1097/MD.0000000000044312)
Supplement: Supplementary file 1 [file medi-104-e44312-s001.docx]

**Table S1. Joint association of physical activity and sedentary behavior with sarcopenia.**

| **Variables** | **Non-sarcopenia**  **(n = 7180)** | **Sarcopenia**  **(n = 689)** | **^a^ Model 1** | | **^b^ Model 2** | | **^c^ Model 3** | |
| --- | --- | --- | --- | --- | --- | --- | --- | --- |
|  |  |  | **OR (95% CI)** | ***P*-value** | **OR (95% CI)** | ***P*-value** | **OR (95% CI)** | ***P*-value** |
| **PA & Sedentary time, n (%)** |  |  |  |  |  |  |  |  |
| Never/Light & Severe | 1194 (16.13) | 145 (24.33) | Reference |  | Reference |  | Reference |  |
| Never/Light & Mild | 1906 (23.12) | 263 (34.31) | 0.98 (0.73, 1.33) | 0.911 | 0.98 (0.73, 1.32) | 0.883 | 0.80 (0.56, 1.13) | 0.203 |
| Moderate & Severe | 695 (11.05) | 68 (12.77) | 0.77 (0.51, 1.15) | 0.196 | 0.76 (0.50, 1.15) | 0.186 | 0.94 (0.61, 1.45) | 0.781 |
| Moderate & Mild | 1072 (15.88) | 112 (14.69) | **0.61 (0.41, 0.91)** | **0.017** | **0.61 (0.41, 0.92)** | **0.018** | **0.58 (0.39, 0.89)** | **0.013** |
| Vigorous & Severe | 946 (14.96) | 30 (3.98) | **0.18 (0.11, 0.28)** | **<0.001** | **0.19 (0.12, 0.30)** | **<0.001** | **0.26 (0.16, 0.42)** | **<0.001** |
| Vigorous & Mild | 1367 (18.86) | 71 (9.92) | **0.35 (0.23, 0.52)** | **<0.001** | **0.39 (0.26, 0.58)** | **<0.001** | **0.41 (0.26, 0.64)** | **<0.001** |

Note: OR, odds ratio; 95 % CI, 95 % confidence interval; PA, physical activity; PIR, family poverty income ratio. Non-normal continuous variables are represented by weighted median (weighted Q25%, weighted Q75%), and categorical variables are represented by n (weighted %).

^a^ Model 1: unadjusted model.

^b^ Model 2: adjusted for age and sex.

^c^ Model 3: adjusted for age, sex, ethnicity, education level, marital status, PIR, alcohol consumption, smoking status, sleep duration, dietary health, self-reported hypertension, self-reported diabetes, and self-reported cardiovascular disease.

**Table S2. Interaction analysis of age and sex in the association between physical activity, sedentary behavior, and sarcopenia.**

| **Variables** | **Age** | **Gender** |
| --- | --- | --- |
|  | **(18-45 years/>45 years)** | **(male/female)** |
|  | ***P*-value^a^** | ***P*-value*^a^*** |
| Sedentary time, h/day | 0.241 | 0.700 |
| Sedentary time, (mild/severe) | 0.331 | 0.503 |
| Total PA, h/week | 0.838 | 0.707 |
| Physical activity, (never/light/moderate/vigorous) | 0.887 | 0.910 |
| PA patterns, (inactive/weekend warrior/regularly active) | 0.798 | 0.369 |

Note: ^a^, *P*-value represents the significance of the interaction analysis, adjusted for age, sex, ethnicity, education level, marital status, PIR, alcohol consumption, smoking status, sleep duration, dietary health, self-reported hypertension, self-reported diabetes, and self-reported cardiovascular disease.

**Table S3**. Associations between physical activity, sedentary behavior, and sarcopenia in PSM analysis, NHANES, 2011-2018.

| **Variables** | **1:1 Matching** | | **1:2 Matching** | | **1:3 Matching** | |
| --- | --- | --- | --- | --- | --- | --- |
|  | **OR (95% CI）** | ***P*-value** | **OR (95% CI）** | ***P*-value** | **OR (95% CI）** | ***P*-value** |
| **Sedentary time, h/day** | 1.03 (0.99, 1.06) | 0.113 | 1.03 (1.00, 1.06) | 0.060 | **1.03 (1.00, 1.06)** | **0.020** |
| **Sedentary time, n (%)** |  |  |  |  |  |  |
| Mild | Reference |  | Reference |  | Reference |  |
| Severe | 0.96 (0.76, 1.21) | 0.722 | 1.02 (0.84, 1.24) | 0.841 | 1.06 (0.87, 1.27) | 0.575 |
| **Total PA time, h/week** | 0.98 (0.96, 1.00) | 0.065 | 0.98 (0.96, 1.00) | 0.063 | 0.98 (0.97, 1.00) | 0.070 |
| **PA, n (%)** |  |  |  |  |  |  |
| Never/Light | Reference |  | Reference |  | Reference |  |
| Moderate | 0.84 (0.64, 1.11) | 0.219 | 0.99 (0.79, 1.25) | 0.929 | 1.04 (0.84, 1.30) | 0.703 |
| Vigorous | **0.70 (0.51, 0.94)** | **0.018** | **0.73 (0.55, 0.95)** | **0.020** | **0.69 (0.53, 0.89)** | **0.004** |
| **PA patterns, n (%)** |  |  |  |  |  |  |
| Inactive | Reference |  | Reference |  | Reference |  |
| Weekend warrior | 0.74 (0.41, 1.32) | 0.302 | 0.87 (0.52, 1.44) | 0.580 | 0.93 (0.57, 1.52) | 0.775 |
| Regularly active | 0.98 (0.75, 1.27) | 0.852 | 1.07 (0.85, 1.35) | 0.576 | 1.07 (0.86, 1.33) | 0.535 |

Note: OR, odds ratio; 95 % CI, 95 % confidence interval; PA, physical activity.
