# Supplementary material for: Impact of physical activity patterns and sedentary behavior on sarcopenia prevalence among adults aged 18 to 59: A cross-sectional study from NHANES
Source: Medicine (Baltimore). 2025 Sep 5;104(36):e44312. doi: 10.1097/MD.0000000000044312 (PMC12419358; doi:10.1097/MD.0000000000044312)
Supplement: Supplementary file 2 [file medi-104-e44312-s002.docx]

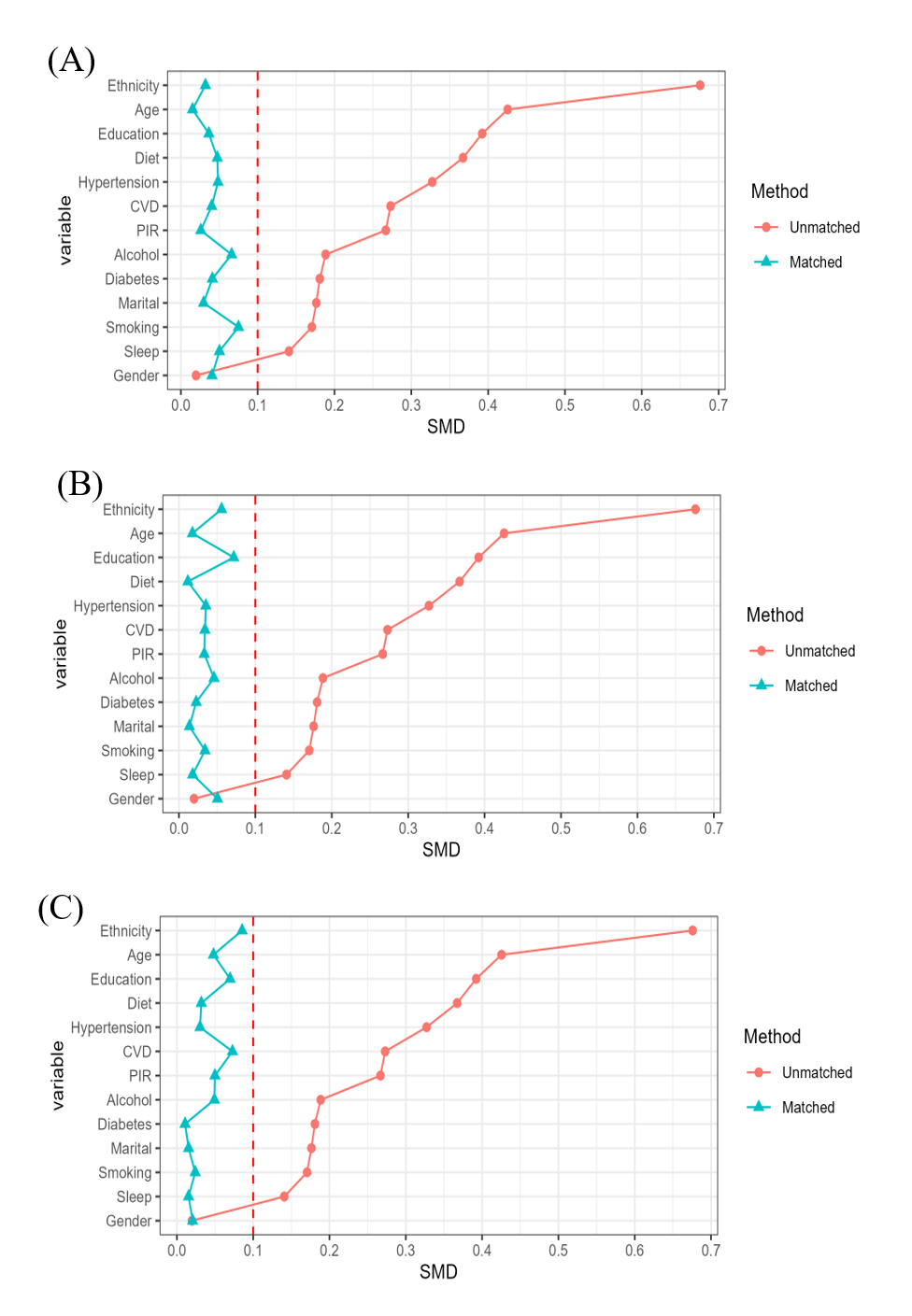


**Figure S1.** The SMD of variables before and after propensity score matching (PSM). (A) 1:1 PSM, (B) 1:2 PSM, and (C) 1:3 PSM. CVD, cardiovascular diseases; PIR, family poverty income ratio; SMD, standardized mean difference.
